# Mental Health of Medical Workers in COVID-19 Pandemic: Restrictions and Barriers

**DOI:** 10.34172/jrhs.2020.16

**Published:** 2020-06-18

**Authors:** Rahim Badrfam, Atefeh Zandifar, Mohammad Arbabi

**Affiliations:** ^1^Department of Psychiatry, Psychosomatic Medicine Research Center, Tehran University of Medical Sciences, Tehran, Iran; ^2^Social Determinants of Health Research Center, Alborz University of Medical Sciences, Karaj, Iran

## Dear Editor-in-Chief

The high transmission power and lethality of COVID-19 disease have led to special attention to the disease and efforts to control it^1^. On the other hand, the COVID-19 pandemic put a wide range of psychological pressure on health care workers. Problems such as depression, anxiety, insomnia, and distress have been reported in many cases^2^. Mental health problems related to health care professionals need proper and comprehensive management during the COVID-19 pandemic^3^.

Following the COVID-19 pandemic, demand for health care staff has increased dramatically. Given the need of society for their effective and permanent presence, it is very important to pay attention to their expected needs^4^. Exposure to physical and mental trauma, high work responsibilities, enduring the loss of patients and colleagues, and the risk of infection are examples of these difficult conditions. For this reason, in addition to facilitating certain conditions for them, as resources needed by society, psychological support for these people is also very important^5^. Identifying their mental health problems and addressing these potential problems is the first step regarding an effective intervention. However, there seems to be a serious limitation in the expression of these problems by health care staff^6^.

“In general, in any biological disaster, fear, uncertainty, and stigmatization themes are common and may act as a barrier to physical and mental health interventions”^7^. We will face a group of personnel who, despite the risk of mental health problems or having some degree of these problems, do not try to improve their conditions. This can pose many challenges for both these individuals and the patients under their care and the related health systems.

In a relatively similar experiment, in the context of Sever Acute Respiratory Syndrome (SARS) epidemics, most staff members were very concerned on becoming infected, although they generally considered this risk to be part of their job situation. About 20% of patients with SARS at that time were health care workers. On the other hand, about half of them experienced social stigmatization and even some of them were, in a way, rejected by the family^8^. In another study followed by the SARS epidemic, both groups of staff, with a history of SARS and no history of SARS, shared a common concern about infecting their families^9^. Although the group that had a history of SARS, thought more about discrimination related to having SARS and other health issues. They saw themselves as more vulnerable to social and occupational discrimination. They also showed a greater prevalence of bone pain, lethargy, and physical weakness, and in addition to attributing some of them to medication side effects, some of these conditions could be attributed to the psychological effects on patients' concerns. As such, there appear to be many barriers to mental health care for staff. Different conditions may also lead to referrals to other medical specialties. In this regard, providing training and creating appropriate awareness of this group of personnel can play an effective role in addressing these issues.

Another important point in this regard is the need for trustworthy behavior and avoidance of denying facts. This can be seen in the sudden spread of COVID-19 in some countries. As we have seen in the Italian experience and historically, we have seen similar conditions during the H1N1 flu pandemic^10,11^. Having confidence in health policy makers can provide the conditions for the delivery of mental health problems without worries and provide the conditions for the improvement of the current situation. Lack of trust can also lead to many concerns, such as feelings of worry about job stability and a lack of proper support. The combination of these issues can increase concerns about the expression of mental problems by staff.

In addition, a very important point is that health care workers sometimes use maladaptive coping strategies^10^. They may deny the matter or consider it insignificant. The use of methods such as self-blame and avoidance can be used by this group of personnel, which can be very worrying. In addition, many normal adaptive coping strategies, such as social communication, exercise, and leisure, have been severely restricted during the COVID-19 pandemic^4^.

Self-efficacy is the other important point in this regard. Defects in self-efficacy which seen among a group of medical workers can be related to the fear of getting sick, and in some of them, it has been associated with post-traumatic stress disorder^9^. Social support was directly related to self-efficacy and was negatively related to stress and anxiety among staff^12^. In staff who lack proper social support, the risk of low self-efficacy is higher. These people are prone to mental health disorders. There are also concerns that they will not raise issues related to mental health^13^. Having the right social support and even using social campaigns at a higher level can be effective in this regard.

Health care providers, especially in the field of COVID-19 pandemics, are at risk for mental health disorders and failure to follow up to manage possible disorders. In order to strive to achieve the right mental health and psychological well-being in health care personnel, especially in the context of chronic stress, it is important to pay attention to creating the right conditions at the individual and organizational levels.

At the individual level, the use of appropriate coping methods such as problem-solving (in what is estimated to be the case under individual control), emotion-based coping (to reduce isolation and increase support), meaning-based coping (for unresolved issues and permanent distress) accompanied with Organizational resilience such as material reserves, back up plans, succession plans and proper management can be effective^14^.

Creating a sense of trust, by meeting the needs of staff for proper personal protection and job stability, is one of the most important ways to express and pursue treatment for possible mental health disorders. The elimination of stigmatization requires public awareness at the community level with the help of social communication tools and efforts to address it at the individual level^15^. This also seems to be helpful to express freely the mental health problems of health care personnel. Proper social support also plays an important role in this regard.

## Conflict of interest

The authors declare that there is no conflict of interest.

## References

[1] Poorolajal J (2020). Neglected major causes of death much deadlier than COVID-19. J Res Health Sci.

[2] Lai J, Ma S, Wang Y, Cai Z, Hu J, Wei N (2020). Factors associated with mental health outcomes among health care workers exposed to coronavirus disease 2019. JAMA Netw Open.

[3] Zandifar A, Badrfam R (2020). Iranian mental health during the COVID-19 epidemic. Asian J Psychiatr.

[4] Gavin B, Hayden J (2020). Caring for the psychological well-being of healthcare professionals in the Covid-19 pandemic crisis. Ir Med J.

[5] Lancet Lancet (2020). COVID-19: protecting health-care workers. Lancet.

[6] Zheng W (2020). Mental health and a novel coronavirus (2019-nCoV) in China. J Affect Disord.

[7] Xiang YT, Yang Y, Li W, Zhang L, Zhang Q, Cheung T (2020). Timely mental health care for the 2019 novel coronavirus outbreak is urgently needed. Lancet Psychiatry.

[8] Koh D, Lim MK, Chia SE, Ko SM, Qian F, Ng V (2005). risk perception and impact of severe acute respiratory syndrome (sars) on work and personal lives of healthcare workers in singapore what can we learn?. Med Care.

[9] Ho SM, Kwong-Lo RS, Mak CW, Wong JS (2005). Fear of severe acute respiratory syndrome (SARS) among health care workers. J Consult Clin Psycholo.

[10] Imai H (2020). Trust is a key factor in the willingness of health professionals to work during the COVID‐19 outbreak: Experience from the H1N1 pandemic in Japan 2009. Psychiatry Cli Neurosci.

[11] Rosenbaum L (2020). Facing Covid-19 in Italy—ethics, logistics, and therapeutics on the epidemic’s front line. N Engl J Med.

[12] Xiao H, Zhang Y, Kong D, Li S, Yang N (2020). The effects of social support on sleep quality of medical staff treating patients with coronavirus disease 2019 (COVID-19) in January and February 2020 in China. Med sci monit.

[13] Bandura A. Cultivate self-efficacy for personal and organizational effectiveness. In Locke EA (editor). Handbook of principles of organization behavior. Oxford: Blackwell; 2000.

[14] Maunder RG, Leszcz M, Savage D, Adam MA, Peladeau N, Romano D (2008). Applying the lessons of SARS to pandemic influenza: an evidence-based approach to mitigating the stress experienced by healthcare workers. Can J Public Health.

[15] Badrfam R, Zandifar A (2020). Stigma Over COVID- 19; new conception beyond individual sense. Arc Med Res.

